# Analysis of NRAS RNA G-quadruplex binding proteins reveals DDX3X as a novel interactor of cellular G-quadruplex containing transcripts

**DOI:** 10.1093/nar/gky861

**Published:** 2018-09-26

**Authors:** Barbara Herdy, Clemens Mayer, Dhaval Varshney, Giovanni Marsico, Pierre Murat, Chris Taylor, Clive D'Santos, David Tannahill, Shankar Balasubramanian

**Affiliations:** 1 Cancer Research UK Cambridge Institute, University of Cambridge, Li Ka Shing Centre, Robinson Way, Cambridge CB2 0RE, UK; 2 Stratingh Institute for Chemistry, University of Groningen, Nijenborgh 4, 9747 AG Groningen, Netherlands; 3 Department of Chemistry, University of Cambridge Lensfield Road, Cambridge CB2 1EW, UK; 4 Bioscience Technology Facility, Department of Biology, University of York, York YO10 5DD, UK

## Abstract

RNA G-quadruplexes (rG4s) are secondary structures in mRNAs known to influence RNA post-transcriptional mechanisms thereby impacting neurodegenerative disease and cancer. A detailed knowledge of rG4–protein interactions is vital to understand rG4 function. Herein, we describe a systematic affinity proteomics approach that identified 80 high-confidence interactors that assemble on the rG4 located in the 5′-untranslated region (UTR) of the NRAS oncogene. Novel rG4 interactors included DDX3X, DDX5, DDX17, GRSF1 and NSUN5. The majority of identified proteins contained a glycine-arginine (GAR) domain and notably GAR-domain mutation in DDX3X and DDX17 abrogated rG4 binding. Identification of DDX3X targets by transcriptome-wide individual-nucleotide resolution UV-crosslinking and affinity enrichment (iCLAE) revealed a striking association with 5′-UTR rG4-containing transcripts which was reduced upon GAR-domain mutation. Our work highlights hitherto unrecognized features of rG4 structure–protein interactions that highlight new roles of rG4 structures in mRNA post-transcriptional control.

## INTRODUCTION

**Figure 1.**
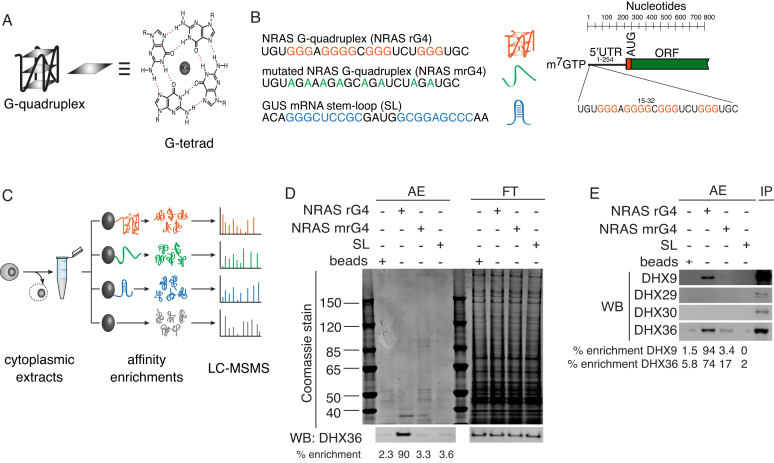
Strategy for affinity enrichment (AE) of proteins interacting with the human NRAS rG4 structure. (**A**) Left, schematic of a G-quadruplex (G4) structure with three stacked G-tetrads (shaded squares) connected by intervening loops (black). Right, drawing of a G-tetrad consisting of four Hoogsten-hydrogen bonded (red) guanines stabilized by a central metal cation (K^+^). (**B**) Left, RNA oligonucleotides used in AEs: top, the NRAS rG4 folded into a G4 structure; middle, mutated NRAS rG4(NRAS mG4) that is unable to form a G4 structure (green indicates Gs mutated to As); and bottom a stem loop (SL, blue indicates hydrogen-bonded stem bases) from the GUS mRNA. Right, location of the rG4 sequence within the 5′-UTR of the human NRAS transcript. (**C**) Workflow for AEs and liquid chromatography-tandem mass spectrometry (LC-MSMS). HeLa cell cytoplasmic extracts were incubated with biotinylated rG4 or control oligonucleotides coupled to streptavidin beads. Affinity-purified proteins were subjected to LC-MSMS for subsequent identification. (**D**) Control AEs of endogenous DHX36. HeLa cell cytoplasmic extracts were incubated with rG4, mrG4 or SL biotinylated oligonucleotides bound to streptavidin beads or with beads alone (beads). Bound protein fractions (AEs) and flow-through (FT) were subjected to SDS-PAGE and stained for total protein (top) or processed for western blotting with a DHX36 antibody (bottom). The presence of DHX36 protein in each lane is presented as a percentage of the signal detected in all lanes below the western blot panel. (**E**) AEs and western blotting for DHX9, DHX29 and DHX30 using antibodies detecting endogenous helicases as described in (D). Input (IP) was 30 μg of cytoplasmic extract. The presence of DHX9 and DHX36 in AEs is presented as a percentage of the signal detected in all lanes below the western blot panel.

Recognition of mRNA secondary structures by RNA binding proteins (RBPs) is essential for post-transcriptional control to influence mRNA processing, stability, transport and translation (1,2). Watson–Crick hydrogen bonding and non-canonical interactions are important in RNA folding, and four-stranded G-quadruplex (G4) secondary structures are key structural features in mRNA (3,4). G4 structures form from guanine (G)-rich sequences in which stacks of G-quartets are stabilized by a central metal cation (Figure 1A). Recently, high-throughput sequencing combined with reverse transcriptase stalling at stabilized RNA G4s (rG4) has revealed over 13 000 loci where rG4 structures form within the human transcriptome *in vitro* (5,6). Evidence supporting rG4 formation in cells includes detection of rG4s in the cytoplasm by immunofluorescence using a G4 structure-specific antibody (7,8). Notably, rG4s are enriched in functionally important regions, including 5′- and 3′-untranslated regions (UTRs) (5,6,9–11).

Several helicases such as DHX36 and DDX21 bind and unwind rG4 structures with pico- or nanomolar affinities (12–14). Another multifunctional helicase is DHX9 which binds several nucleic acid secondary structures including G4s but with a preference for RNA substrates (14). Thus, cells possess specialized enzymes that recognize and resolve rG4s which may be important for post-transcriptional processes such as mRNA translation, transport or stability.

rG4s have been functionally implicated in several neurodegenerative diseases, such as amyotrophic lateral sclerosis (ALS), frontotemporal dementia (FTD) and Fragile X syndrome (FXS) (15,16). The underlying cause of FXS is a CGG-rich repeat expansion in the FMR1 gene that contributes to protein silencing due to rG4-mediated translational inhibition (17). Likewise, ALS is defined by a GGGGCC repeat expansion in C9orf72, which leads to a repeat-length-dependent accumulation of aborted rG4-containing transcripts (8). It has been proposed that rG4s have roles in cancer development and progression as several 5′-UTRs of oncogene mRNAs are enriched in rG4s (5,10,11,18,19). The presence of a 5′-UTR rG4 hampers cap-dependent translation of several oncogene messages including NRAS and BCL2 *in vitro* (19–21).

As rG4s frequently occur in mRNAs and have important regulatory roles, comprehensive identification of cytoplasmic rG4-interacting proteins is needed to dissect rG4 function. We have therefore used an unbiased affinity proteomics approach to catalog cytoplasmic interactors of the human NRAS 5′-UTR rG4. This rG4 was selected due to the relevance of NRAS in tumorigenesis (22). Moreover, folding of the NRAS rG4 into a stable parallel intramolecular G4 is well-characterized biophysically and this rG4 has been shown to inhibit translation *in vitro* (20). Herein, we identify cytoplasmic rG4-interacting proteins that have not previously been demonstrated to interact with an rG4 structure. Notably, over half of the rG4 interactors contained a glycine-arginine-rich (GAR) domain, and we show that this is required for the NRAS rG4-DDX3X interaction. This interaction was recapitulated by transcriptome-wide individual-nucleotide resolution UV-crosslinking and affinity enrichment (iCLAE) in cells. Overall, our work highlights the utility of identifying rG4-interacting proteins to generate mechanistic insights into rG4-mediated post-transcriptional control.

## MATERIALS AND METHODS

### Materials

Anti-MYC, Hemagglutinin tag, DHX36, DDX5, DDX17, DHX9, DHX29 and DHX30 antibodies were purchased from Abcam, the V5-tag antibody was obtained from Source BioScience, DDX3X antibody was ordered from Santa Cruz and FXR1 antibody was purchased from Cell Signaling. RNA oligonucleotides were ordered from Integrated DNA Technologies. Streptavidin magnetic beads were obtained from Promega and Strep-Tactin magnetic nanobeads were purchased from IBA.

### Plasmids

cDNAs of rG4 interactors were purchased from various commercial sources (Supplementary Table S3) and were inserted into the pDONR™221 entry vector (ThermoFisher) using specific primers (Supplementary Table S4). Alternatively, RNA from HeLa cells was converted into cDNA using SuperscriptIV (ThermoFischer). Constructs that produce N-terminally or C-terminally tagged proteins were prepared by recombination of pDONR™221 entry clones with pcDNA™3.1/nV5-DEST (ThermoFisher) or pCS2–6myc-GW (23). To generate stable Doxycycline-inducible cell lines expressing tagged rG4-interactors, pDONR™221 entry clones were recombined with the pcDNA5/FRT/TO/SH/GW destination vector (23). RG/RGG motif mutations were introduced by generation of overlapping fragments using corresponding primers (Supplementary Table S4).

### Cell lines

HeLa (ATCC) and Flp-In T-REx 293 cell line (ThermoFisher) were cultured in Dulbecco’s modified Eagle’s medium (DMEM, Sigma) with 10% fetal bovine serum (FBS) (Gibco) or Tetracycline-free FBS (Clontech), respectively. Cells were mycoplasma tested and genotyped. Stable Flp-In T-REx 293 cell lines expressing selected Strep-tag (ST)/HA affinity tagged rG4-interactors (23) were generated by transfecting destination vectors into cells following the manufacturer's instructions (ThermoFisher). Cells were selected with Blasticidin (Gibco) and Hygromycin D (Sigma). Single clones were tested for Doxycycline inducibility.

### Transfections, cell lysates, western blot and Wes Simple Western analysis

HeLa cells were transfected at a confluency of 1 × 10^6^ cells with 5 μg of plasmid using TransIT-LT1 Transfection Reagent (Mirus Bio LLC). After 45 h, cells were lysed using hypotonic lysis buffer with 0.5% Sodium deoxycholate, 0.5% TritonX100, 2 mM Dithiothreitol (DTT) as previously described in (24). For western blot analysis 30 μg of hypotonic extract or 25 μl of 50 μl affinity enrichments (AEs) in Laemmli sample buffer (Sigma) were loaded 12% sodium dodecyl sulphate-polyacrylamide gel electrophoresis (SDS-PAGE) gels (ThermoFisher) and proteins were transferred to a nitrocellulose membrane using the iblot2 system (ThermoFisher). Membranes were blocked with Odyssey Blocking Buffer (LI-COR) and incubated with the first antibody followed by a second, fluorophore conjugated antibody (LI-COR). Fluorescent images were established using the Odyssey CLx (LI-COR) and quantification of bands was performed with Fiji image analysis software. Briefly, affinity enriched protein bands were quantified by marking all bands individually with rectangular sections in the gray scale image. The Fiji software then plots peaks representing the intensity of the band in the selected areas. Each peak was expressed as percentage of the total size of all selected bands. Capillary electrophoresis in a Wes Simple Western System (ProteinSimple) was performed as previously described by the manufacturer (Proteinsimple (https://proteinsimple.com)). The Wes immunoassay is a capillary-based system where samples are loaded into the capillary automatically and separated by size as they migrate through a stacking and separation matrix. The separated proteins are then immobilized to the capillary wall via proprietary, photoactivated capture chemistry. Target proteins are identified using a primary antibody and immuno-probed using a horseradish peroxidase (HRP) conjugated secondary antibody and a chemiluminescent substrate. The resulting chemiluminescent signal is detected and quantified. Wes analysis was performed with 1 or 5 μg of hypotonic extract or 2.4 μl of 50 μl AEs in Laemmli sample buffer. Each western blot or ‘Wes’ Simple Western experiment is a representative of three independent experiments.

### AEs and liquid chromatography-tandem mass spectrometry (LC-MSMS)

AEs were performed as previously described with minor alterations (25). Prior to AEs, 10 μM biotinylated oligonucleotides were folded in 1 x phosphate-buffered saline supplemented with 2M KCl by boiling for 5 min followed by cooling to room temperature. Roughly, 1000 μg of cytoplasmic cell lysate were used for AEs combined with 50 μl of slurry streptavidin magnetic beads (Promega) that were previously bound to folded, biotinylated oligonucleotides. AEs were performed at 4°C for 3 h in RNA-pull-down buffer (20 mM Hepes, pH 8, 100 mM NaCl, 20% v/v glycerol, 0.2 mM ethylenediaminetetraacetic acid (EDTA), 1 mM DTT, 0.01% Nonidet-P40, 50 μg/mL yeast tRNA (Ambion), 160 U/ml RNasin). Magnetic beads were washed three times with RNA-wash buffer (20 mM Hepes, pH 8, 100 mM NaCl, 20% v/v glycerol, 0.2 mM EDTA, 1 mM DTT, 0.01% Nonidet-P40). Two biological replicates were analyzed by LC-MSMS as previously described (26) after on-bead trypsin digestion of affinity captured proteins. Raw data were processed using Proteome Discoverer (v1.4) and Mascot and/or SEQUEST as search engines.

### iCLAE

Procedures were performed as previously described (27) with the following alterations. Two 10 cm plates of Flp-In T-REx 293 cells expressing wild-type (WT) or four 10-cm plates for RG mutant DDX3X were seeded at a density of 5 × 10^6^ and protein production was induced over night with 0.01 μg/ml Doxycycline. RBP–RNA interactions were stabilized by UV crosslinking (254 nm, 200 mJ/cm^2^), followed by lysis in hypotonic lysis buffer. Cytoplasmic lysates were replenished to a final concentration of 50 mM Tris–HCl (pH 7.4) 100 mM NaCl and 0.1% SDS. Subsequently, RNAse/DNAase digestion was performed as previously described (27). AEs utilizing the Strep-tag on DDX3X and RG mutant DDX3X were performed by incubating lysates with Strep-Tactin magnetic beads for 3 h at 4°C. Reverse transcription (RT) was performed in G4 optimized lithium RT buffer (5).

### RNA-seqencing (RNA-seq)

Cells were grown in DMEM 10% FBS to 70% confluency. Protein expression of DDX3X WT and RG mutant was induced with 0.01 μg/ml Doxycycline. RNA was extracted using TRI^®^ reagent (Sigma-Aldrich) according to manufacturer’s instructions. RNA-seq libraries were generated using the Illumina Truseq Stranded total RNA kit (Illumina, cat #RS-122–2301) as per the manufacturer’s instructions and sequenced using the Illumina NextSeq 500 kit.

### Bioinformatic analysis of AE-LC-MSMS data

Significance analysis of NRAS rG4 interactors was performed by using the edgeR package in RStudio (http://www.rstudio.org/): peptide counts from the AE-LC-MSMS data were fitted with the generalized linear model (glmFit) using library size only as normalization, and *P*-values and false discovery rate (FDR) were estimated from the differential signal analysis (glmLRT) by contrasting NRAS rG4 counts to the merged set of negative controls (NRAS mrG4, SL and beads-only). A list of high-confidence interactors was created by selecting only proteins with FDR < 0.05. To represent rG4 interactors in a network, high confidence interactors were imported into Cytoscape version 3.5.1 (www.cytoscape.org) (28) and intersected with physical interactions imported from IMEX-complying databases using PSICQUIC Universal Client app. Functional modules within the network were identified with the MCODE 1.4.2 app (http://baderlab.org/Software/MCODE) and gene ontology annotations were added using the ClueGO 2.3.3 app (http://www.ici.upmc.fr/cluego/cluegoDescription.shtml). Term enrichment was performed by right-sided hypergeometic test with a Benjamini–Hochberg corrected *P*-value.

### RNA-seq and iCLAE analysis

Raw sequencing files for RNA-seq libraries were preprocessed using cutadapt to remove sequencing adapters and low quality sequencing tails (options –q 10). Trimmed files were aligned to the human genome (*GRCh37/hg19*) using tophat2 and using the UCSC gtf file provided by Illumina iGenomes as an annotation file (http://support.illumina.com/sequencing/sequencing_software/igenome.html). Gene counts were calculated using htseq-count and the same gtf file. Differential expression analysis was done using the R package edgeR. Isoform quantification was performed using the cufflinks software (https://github.com/cole-trapnell-lab/cufflinks) and the value for the same condition (WT DDX3X, RG-mutant DDX3X and Negative) were averaged. Transcripts with average FPKM of at least 0.1 in any condition were considered as expressed, and those mapping to protein coding or lncRNA from the gencode version 19 annotation (*n* = 24 590) were then used to assemble the expressed transcriptome fasta file for the following iCLAE analysis. Identical reads of iCLAE-seq libraries were removed and de-multiplexed according to their 4-nt pattern sequence at the 5′-end of each read (e.g. N3-GGTT-N2). Libraries were then pre-processed with cutadapt to remove 3′ sequencing adapters and low quality sequencing tails. Highly repetitive reads, i.e. those having at least 10 equal nucleotides (e.g. A(58), T{10,n}, etc.), were removed and aligned to the *hg19* version of the human genome using bwa mem (http://bio-bwa.sourceforge.net/). After alignment, reads with mapping quality (MAPQ) < 10 were removed and those aligning to the same position while also having the same barcode were eliminated, as they constitute most likely polymerase chain reaction duplicates. Coverage files were calculated (bedtools), regions with signal above 10 read counts extracted and intervals closer than 30 nt were merged into a single peak region. Merged peak regions less than 30 nt in width were removed. Next, only reads aligning with MAPQ ≥ 10 with expressed transcripts (bwa-hg19 aligned bam files) were further mapped to the expressed transcriptome RSEM (https://github.com/deweylab/RSEM). Coverage transcript files were calculated and normalized for the total estimated count in each iCLAE library, and peaks were called. One hundred base pairs flanking the middle of a peak were considered as binding regions of peaks below 100 nt and sequences from these regions were extracted with bedtools. UTRs and coding sequence (CDS) analyses was performed by considering the same gencode version 19 annotation file as described above (https://www.gencodegenes.org/releases/19.html). Fold enrichment analysis was performed by randomly shuffling of peaks throughout expressed transcripts (bedtools shuffle). To describe the overlap with published datasets fold enrichment was calculated similarly. G4 motif analysis was performed by considering the following regular expression G_2+_ N_1–12_ G_2+_ N_1–12_ G_2+_ N_1–12_ G_2+_, summarized as (G2-L12)_4_.

## RESULTS

### Identification of cytoplasmic NRAS rG4-interacting proteins

We applied an unbiased proteomics approach to identify cytosolic proteins that interact with the NRAS 5′-UTR rG4 structure. Biotinylated oligonucleotides containing the rG4 sequence found in the 5′-UTR of NRAS were folded into a rG4 structure (see ‘Materials and Methods’ section). Folded rG4 and control oligonucleotides (Figure 1B) were immobilized on streptavidin beads and used as baits for affinity enrichments (AEs) of proteins from HeLa cell cytosolic extracts (Figure 1C) (25). To critically evaluate specific rG4 interactors, a G-to-A mutated NRAS sequence (mrG4) that is unable to fold in to a G4, a stem-loop-forming sequence (SL), and empty beads (beads i.e. no oligonucleotide) were used in independent AEs (Figure 1B). The integrity of rG4 formation and the failure to form a G4 structure in the mutated control was confirmed *in vitro* using circular dichroism spectroscopy (CD) and UV thermal melting analysis (Supplementary Figure S1). Using an anti-DHX36 antibody, we confirmed strong enrichment of DHX36, a well-known rG4 interactor, in the NRAS rG4 (90%) AEs compared to the mrG4 (3.3%), SL (3.6%) and bead (2.3%) controls, which validated our approach (Figure 1D). Intriguingly, assessment of selected DHX helicase family members using antibodies recognizing endogenous proteins showed that the NRAS rG4 interacted selectively with DHX9 (94%) compared to controls (mrG4 3.4%, SL 0%, beads 1.5%) but did not enrich for DHX30 or DHX29 (Figure 1E). These data suggest high specificity and selectivity of our assay.

**Figure 2.**
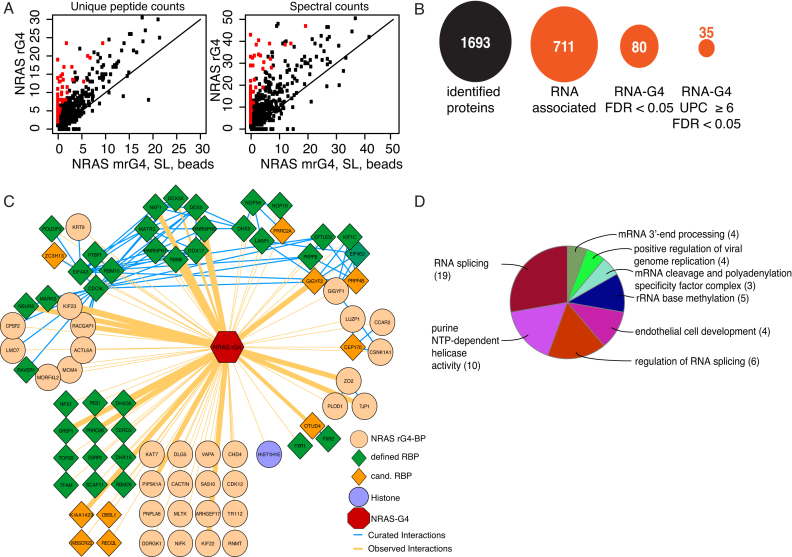
High-confidence proteins interacting with the NRAS rG4 structure. (**A**) Scatter plot of average unique peptide counts (UPCs) (left), and average spectral counts (right), comparing NRAS rG4 AEs relative to controls (mrG4, SL and beads) (Supplementary Figure S2 and Table S2). Each dot represents one protein. The X-axis shows the average of peptide (or spectral) counts for mrG4, SL and beads combined (six replicates). The Y-axis represents the average of peptide (or spectral) counts for NRAs rG4 (two replicates). Proteins in red are significantly enriched. (**B**) Overview of filtering parameters to identify high-confidence interactors; false discovery rate (FDR) (**C**) Cytoscape analysis of 80 high-confidence NRAS rG4 interacting proteins. Green diamonds represent previously identified mRNA binding proteins, dark orange diamonds represent candidate mRNA binding proteins and light orange circles represent novel rG4 interactors not previously known as a mRNA-binding proteins. The width of orange lines (edges) describes the probability (‘FDR.G4_vs_rest’ Supplementary Table S2, sheet ‘FDR > 0.05’) of interaction with the NRAS rG4-binding proteins. Blue edges between high confidence interactors are previously published interactions. (**D**) Gene ontology analysis pie chart of significantly enriched functional groups for identified high-confidence rG4 interactors. Figures in brackets are the number of proteins in each group.

Next, proteins bound to the rG4 oligonucleotides and control samples were subjected to on-bead tryptic digestion followed by LC-MSMS (26). We chose this qualitative approach due to its simplicity, cost effectiveness, time effectiveness and the relatively low requirement for bioinformatic analysis as compared to stable isotope labeling by amino acids in cell culture (SILAC) (29). Two biological replicates showed high reproducibility, as judged by dot plots comparing unique peptide counts (UPCs) from each replicate (*r* = 0.67 - 0.85) (Supplementary Figure S2). Overall, 711 proteins interacted with RNA (rG4, mrG4 or SL but not beads). NRAS rG4-specific interactors were then identified by comparing UPCs of rG4 interactors to controls (CTRs: mrG4, SL and beads) using a linear fit model (Figure 2A and B; Supplementary Table S1). These were then ranked according to their false discovery rate (FDR) (significant FDR < 0.05, column ‘FDR.G4_vs_CTRs’ Supplementary Table S1) which revealed 80 significant rG4 interactors. A more refined list was curated by only considering proteins with 6 or more UPCs, resulting in 35 rG4-specific proteins (column ‘G4.av ≥ 6’ Supplementary Table S2). These strict selection criteria identified novel and previously characterized rG4-interacting proteins, including DHX36 (ranked 5) and DHX9 (ranked 31).

Functional relationships within the primary list of 80 NRAS rG4 interactors were determined using Cytoscape, which generates a network of physical relationships (edges) between the interactors (nodes) (28,30) and their probability of interaction with the NRAS rG4 bait (Supplementary Tables S1 and 2; Figure 2C). Known and predicted candidate RBPs (green and orange diamonds, respectively, Figure 2C) were then overlaid on to this network (31,32). Notably, the majority of NRAS rG4 interactors have previously been annotated as RBPs (48 proteins, 60%). However, 32 proteins (orange circles) were not known as RBPs. Highly connected nodes were identified computationally (33), which revealed several interconnected complexes such as the HRNPH1/DDX5/DDX17 complex previously described to influence rG4-dependent splicing (34). Another complex not previously linked to rG4-mediated mechanisms was the eIF4E2/GIGYF1/GIGYF2 complex, which has a role in the negative regulation of translation initiation during development (35). Gene Ontology enrichment analysis was then performed to test which functional categories were over-represented in the rG4 interactor dataset (36) (Figure 2D and Supplementary Figure S3). Of 80 high-confidence rG4 binders, 55 were assigned to specific terms/pathways, including splicing (19 proteins), purine NTP-dependent helicase activity (10 proteins), regulation of splicing (6 proteins) and RNA biogenesis (polyadenylation, cleavage and 3′-end processing, 7 proteins). Strikingly, rRNA base methylation was the most significantly enriched term, with NSUN5, an rRNA methylase (37), being ranked the fourth most significant interactor (Supplementary Table S2). Overall, our approach has identified new rG4-interacting proteins giving insights into previously unknown and unexpected functions for rG4s and their binding partners.

### Validation of identified NRAS rG4 interactors

**Figure 3.**
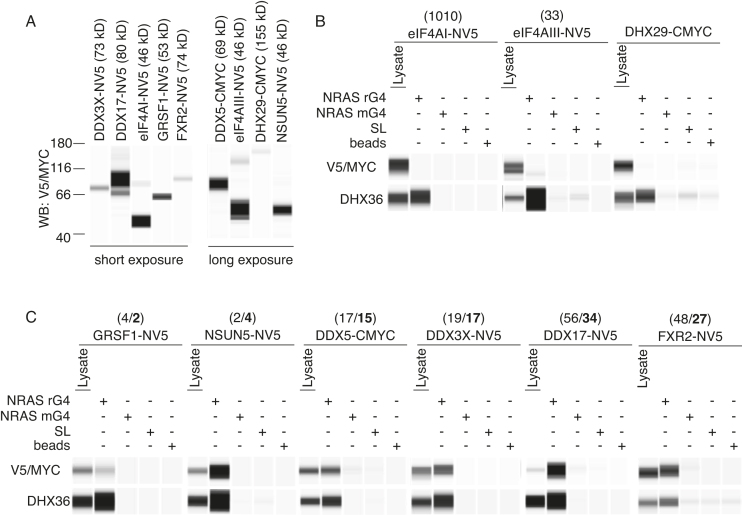
Validation of novel NRAS rG4 high-confidence interactors. (**A**) Expression of epitope-tagged rG4-interacting proteins in HeLa cells followed by Wes Simple Western analysis for indicated tags, NV5 (N-terminal tag) or CMYC (C-terminal tag). A total of 1 μg of cytoplasmic lysate was loaded. (**B** and **C**) AEs for indicated proteins from cytoplasmic HeLa cell extract as per Figure 1 using rG4, mrG4 and SL oligonucleotides or bead only baits followed by Wes Simple Western analysis detecting the epitope tag. As positive control AEs of endogenous DHX36 is shown below each blot. Numbers on top of indicated proteins show ranking of the respective protein according to Supplementary Table S1 (FDR < 0.05; 80 interacting proteins) or in bold according to Supplementary Table S2 (FDR < 0.05 UPC ≥ 6; 35 interacting proteins). A total of 5 μg of cytoplasmic lysate was loaded.

To validate the rG4-binding proteins identified, selected proteins were epitope-tagged with either N-terminal V5 (NV5) or C-terminal MYC (CMYC) and AEs were performed with rG4 or control baits followed by Wes Simple Western analysis to evaluate rG4–protein interactions. Candidates were chosen based on the ranking of the proteins (Supplementary Table S2) and potential links to G4-mediated control mechanisms. Hence, cytoplasmic actin/tubulin transport affiliated proteins (kinesins KIF22 and KIF23), or kinases (MARK3, CDK12), or NFX1, which is a general nuclear-cytoplasmic RNA export factor (38), were excluded for this study. For similar reasons, the GIGYF1/GIGYF2/eIF4E2 complex, which inhibits translation initiation, was not studied (35). We focused on DDX17 and DDX5, each of which has a reported G4-relevant role in splicing (34), but their role in post-transcriptional control is not well explored. DDX3X is a DEAD box helicase related to DDX17 and DDX5 but there is no previous report of a DDX3X–rG4 interaction. Further candidates selected for validation were FXR1 and FXR2, which are homologs of the fragile X mental retardation protein (FMRP) that is known to bind to G4s (39). While the expression of several tagged proteins could be confirmed (Figure 3A), RBM6 (Rank 1) and PRRC2B (Rank 2) could not be transiently expressed (data not shown). Endogenous DHX36 binding to the NRAS rG4 but not to control RNAs was used as a positive control (Figure 3B and C). As endogenous DHX29 did not interact with any baits (Figure 1E), tagged DHX29 was used as a negative control and showed no binding confirming that the epitope tag does not independently interact with oligonucleotide baits (Figure 3B).

Using this approach, we confirmed GRSF1, NSUN5 and FXR2 as rG4-binding proteins that had no or minimal binding to mrG4, SL or B controls (Figure 3C). Likewise, we determined the DEAD box helicase DDX3X as a novel rG4 interactor and that the DDX5 and DDX17 helicases were also specifically enriched by the NRAS rG4. Previously, eIF4AI, another DEAD box RNA helicase pivotal for translational initiation, was shown to unwind rG4 structures (19). In our AE-LC-MSMS experiments eIF4AI was not a significant interactor (ranked position 1102, Supplementary Table S1) and tagged eIF4AI expression did not reveal any interaction with the NRAS rG4 bait (Figure 3B). eIF4AIII, another helicase closely related to eIF4AI (40), but not known to interact with rG4s, also showed lower binding to NRAS rG4 as compared to DHX36 (Figure 3B), despite being ranked 32 in the high confidence interactors. Together, our experiments confirmed specific interaction of the NRAS rG4 with several identified high-confidence interactors.

### Differential selectivity for rG4 structures by NRAS rG4-binding proteins

**Figure 4.**
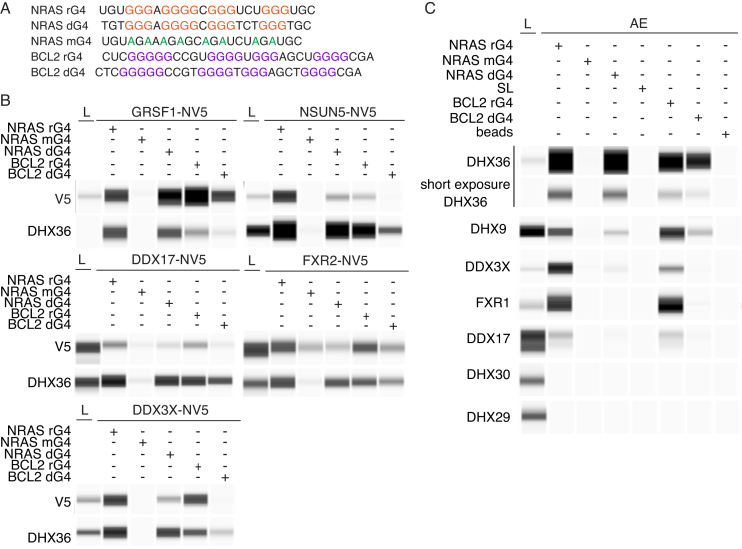
Differential G4 selectivity of selected high-confidence rG4 interactors. (**A**) Sequences for RNA (rG4/mG4) or DNA (dG4) G4 oligonucleotides. Red or purple letters indicate tetrad Gs. Shown in green are G to A mutations in the NRAS rG4 sequence which prevent G4 folding. (**B**) AEs and Wes Simple Western analysis for expressed-tagged proteins were performed as described in Figures 1 and 3 with endogenous DHX36 used as a positive control. A total of 1 μg of cytoplasmic cell lysates (L) loaded in GRSF1, NSUN5 and DDX3X panels while 5 μg were loaded in the DDX17 and FXR2 panels. (**C**) as (B) but using antibodies against selected endogenous proteins.

RBPs can bind several mRNA targets and, in some cases, can interact with DNA through cytoplasmic-nuclear shuttling to execute different functions (1,41). We therefore explored the binding selectivity of identified NRAS rG4-interacting proteins for the 5′-UTR rG4 (BCL2) and for DNA G4 versions of the NRAS and BCL2 sequences. The folding of oligonucleotides into G4s was confirmed by CD spectroscopy and UV thermal melting spectroscopy (Supplementary Figure S1). Proteins were affinity-enriched with either RNA or DNA oligonucleotides containing either the NRAS or BCL2 G4 structures (Figure 4A). Endogenous DHX36 served as a positive control. DHX36 exhibited an apparent preference for NRAS over BCL2 oligos. When RNA or DNA forms of NRAS or BCL2 were compared, RNA oligos appeared to preferentially bind DHX36 (Figure 4B). Each of the affinity-tagged NRAS rG4-interacting proteins, GRSF1, NSUN5, DDX3X, DDX17 or FXR2 showed an individual qualitative preference for different G4s (Figure 4B), with a general preference for rG4s over DNA G4s and for NRAS over BCL2. Notably, NSUN5 appeared to show significant selectivity for the NRAS rG4.

We next evaluated endogenous rG4–protein interactions by immuno-detection using specific antibodies (Figure 4C). The binding specificity of tagged DDX3X (Figure 4B) was accurately recapitulated by the endogenous DDX3X protein (Figure 4C). Results for endogenous and epitope-tagged DDX17 also suggest an interaction with rG4 structures. Our results show that endogenous DHX9, a known rG4 binder (13), preferentially interacted with the NRAS/BCL2 rG4s when compared to DNA G4s. Comparable to its tagged homolog FXR2, endogenous FXR1 was also seen to associate with rG4s but not DNA G4s, while GRSF1 binds all G4s tested. Endogenous DHX9 and DHX30 showed no evidence of G4 interaction (Figure 4C). Together, these data indicate that identified rG4 interactors have a preference for selected rG4s over the corresponding DNA versions.

### Glycine-arginine domains are enriched in NRAS rG4 interactors

**Figure 5.**
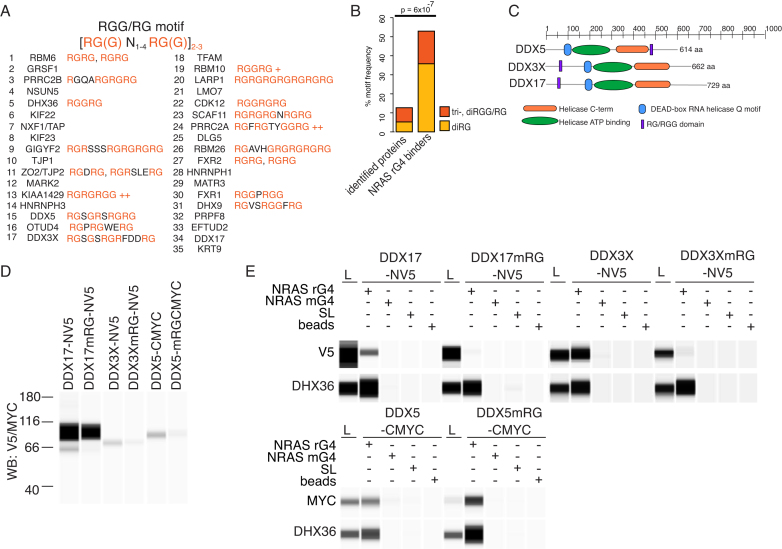
GAR domains are common to high-confidence rG4 interactors and are important for rG4 interactions. (**A**) Occurrence of tri-, diRGG/RG or diRG motifs in the 35 high confidence NRAS rG4 interactors. (**B**) Statistical analysis of tri-, diRGG/RG or diRG motif frequency compared to background. ‘Identified proteins’ are all AE-LC-MSMS identified proteins, proteins in ‘beads only' condition subtracted. NRAS G4 binders represent the top 35 high confidence rG4 interactors (FDR < 0.05 UPC ≥ 6, Supplementary Table 2). (**C**) Overview of DDX5, DDX3X and DDX17 showing positions of tri-, diRGG/RG or diRG motifs and indicated domains. (**D**) Expression of DDX5, DDX3X and DDX17 and GAR-mutant versions (mRG) in HeLa cells detected by Wes Simple Western analysis for the indicated epitope tag. A total 1 μg of cytoplasmic cell extract was loaded. (**E**) AEs and Wes Simple Western analysis for expressed, tagged proteins and their GAR-mutant counterparts were carried out as for Figures 1 and 4 with endogenous DHX36 used as a positive control. A total of 1 μg of cytoplasmic extract was loaded.

GAR domains are comprised of RGG and/or RG repeats and are important features in rG4 binding (42). Therefore, we calculated the presence of di/tri-RGG and di/tri-RG motifs in 35 most significant NRAS G4 interactors described above. In total, 55.8% of the 35 NRAS rG4-binding proteins contained di-RGG/tri-RG (38.2%) or di-RG (17.7%) domains. This contrasts with only 5.2% di-RGG/tri-RG or 8.0% di-RG motifs detected in the entirety of proteins identified by AE-LC-MSMS (NRAS rG4, mrG4, SL and beads) (Figure 5A and B).

Next, we explored whether binding of selected proteins, DDX3X, DDX5 and DDX17, (Figure 5C) to the NRAS rG4 structure is dependent on GAR domains by mutating certain arginines in the RG/RGG domain to alanine (Supplementary Tables S1, 3 and 4). Affinity-tagged versions of WT and mutant proteins (mRG) were expressed in HeLa cells (Figure 5D) and the binding to rG4 oligonucleotides and controls tested (Figure 5E). RG/RGG domain mutation of DDX3X and DDX17 substantially abrogated binding to the NRAS rG4 bait (Figure 5E). By contrast, mutation of the DDX5 GAR domain did not disrupt rG4 binding indicating that another domain in the protein must facilitate binding.

### The GAR domain in DDX3X mediates interaction with rG4-containing mRNAs in cells

**Figure 6.**
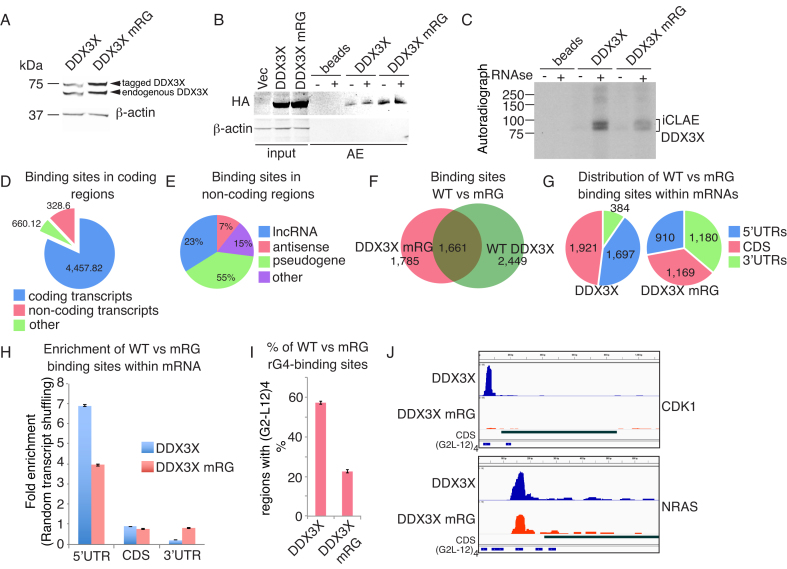
DDX3X mRNA binding is enriched at G4 sites in 5′-UTRs and depends on the presence of an intact GAR domain. (**A**) Western blotting showing expression of tagged WT DDX3X and RG-mutant (mRG) DDX3X protein in FLP-In T-Rex293 cells after Doxycycline induction for 24 h. DDX3X WT and RG-mutant DDX3X are expressed at similar levels to endogenous DDX3X, detected using an antibody against DDX3X. (**B**) Western blot confirming AEs of tagged WT and RG-mutant DDX3X using anti-HA antibodies for detection. Western blotting was performed on affinity-purified material that was used for the iCLAE procedure. (**C**) Autoradiograph of WT and RG-mutant DDX3X complexed with radiolabeled cellular RNA before (−) and after RNAse (+) treatments. (**D**) Distribution of WT DDX3X peaks using Gencode (v19) annotation. (**E**) Distribution of WT DDX3X peaks within non-coding transcripts. Other denotes rRNA, snRNA and miscellaneous RNAs. (**F**) Venn-diagram showing the overlap and unique peaks for WT and RG-mutant DDX3X proteins after transcriptome alignment. (**G** and **H**) Distribution (G) and enrichment (H) of WT and RG-mutant DDX3X proteins. Peak enrichment calculated following random shuffling of peaks in the expressed transcripts. (**I**) Percentage of occurrence of the G4 motif (G2-L12)_4_ within WT and RG-mutant DDX3X. ( **J**) Genome browser view of NRAS and CDK1 mRNAs. iCLAE tracks show peaks of WT or RG-mutant of DDX3X. The CDS tract describes the open reading frame of the plotted transcript and underneath the predicted (G2-L12)_4_ motives within the transcripts were added.

Our AE-LC-MSMS experiments revealed DDX3X as a new rG4-interacting protein. DDX3X is implicated in several aspects of RNA biology and mutations in DDX3X are linked to tumorigenesis, especially medulloblastoma (43,44). Thus, we aimed to identify whether DDX3X interacts specifically with endogenous transcripts containing rG4s. Importantly, in contrast to earlier individual-nucleotide resolution UV-crosslinking and immunoprecipitation (iCLIP) experiments (45,46), our approach was based on the antibody free Strep-tag—Streptactin system for AEs (see ‘Materials and Methods’ section). Furthermore, the method was adapted to enhance the recovery of rG4 targets by comparing WT DDX3X with the rG4-binding impaired RG-mutant together with protocol enhancements to recover G-rich rG4 motifs (see ‘Materials and Methods’ section). Hence, we performed individual-nucleotide resolution UV-crosslinking affinity enrichments (iCLAE) using HEK293 cells expressing Strep-tag/haemaglutinin (ST/HA)-tagged WT or RG-mutant DDX3X proteins at endogenous levels (Figure 6A) (27,47). Next, cells were UV irradiated to cross-link RNAs to WT or RG-mutant DDX3X followed by isolation of cytoplasmic RNA–protein complexes. After RNAse treatment and RNA end-labeling, ST/HA-tag AEs recovered similar amounts of WT and RG-mutant DDX3X protein (Figure 6B), but less RNA was obtained from the RG-mutant compared to WT DDX3X (Figure 6C). iCLAE libraries were prepared using a lithium buffer for reverse transcription to prevent polymerase stalling at G-rich regions (5). To improve recovery of the reduced RNA binding by the RG-mutant, an increased number of cells was used as starting material in this case. No library was amplified from the ‘beads only' control (Supplementary Figure S4). iCLAE and total RNA sequencing reads were aligned to the human genome (*hg19*) and peaks were called for regions with ≥ 10 reads with a maximum allowed gap of 30 nt (see ‘Materials and Methods’ section).

Overall, 5443 WT DDX3X peaks were identified in two out of three biological replicates, which corresponds with previously published iCLIP datasets (Supplementary Figure S5A–C). The majority of peaks (4457, 82%,) aligned within coding transcripts, 12% (660) to non-coding regions and 6% (328) to intergenic regions (Figure 6D). Most non-coding peaks (55%) were annotated as pseudogenes including long non-coding (23%) and antisense RNAs (7%), while the remainder mapped to rRNA, snRNA and other miscellaneous RNAs (Figure 6E). To rule out any bias from non-specific binding due to transcript abundance, we evaluated the correlation between gene expression levels and iCLAE signal for WT DDX3X protein (Supplementary Figure S6A and B). There was little correlation (*r* = 0.29) between transcript levels and WT DDX3X binding. As the DDX3X-specific iCLAE signal was primarily found in coding transcripts, we re-aligned the reads to the human coding transcriptome. This revealed that mutation of the GAR domain significantly altered the binding properties of DDX3X resulting in a reduced peak count (3446) with 48% overlap with the WT protein. (Figure 6F, see ‘Materials and Methods’ section and Supplementary Table S5). Of the 4110 WT DDX3X-specific peaks, 1697 were located within the 5′-UTRs (∼6-fold enrichment) and 1921 in coding exons (∼3-fold enrichment) (Figure 6G). Furthermore, an altered binding site preference compared to WT DDX3X was detected when WT DDX3X and the RG-mutant were compared (Figure 6H).

To investigate potential binding targets, a motif analysis was performed in which 100 nt around the center of peaks, located within annotated transcripts, were scanned for the presence of the (G2-L12)_4_ G4 motif (Figure 6I). Strikingly, the G4 motif was found in 55% of unique WT DDX3X peaks. Even though more cells were used to obtain a library in the DDX3X RG-mutant condition, only 23% of peaks contained the G4 motif (*P* = 3.4e-112 with the Chi-square test for proportions; method prop.test() in the statistical software R). The impaired rG4-binding ability of the RG-mutant DDX3X is consistent with our AE experiments that showed that the RG-mutant DDX3X protein is not captured by the NRAS rG4 bait (Figure 5E). MEME motive-based sequence analysis revealed that DDX3X binding sites that do not contain an rG4 defined by (G2-L12)_4_ still contained G-rich sequence motifs (motif 1, 4 and 5 in Supplementary Figure S7A). There is potential for these G-rich regions to form non-canonical G-quadruplexes. Interestingly, the non-rG4 binding RG-mutant shows enrichment for A-rich motifs (motive 2 and 6, Supplementary Figure S7A), perhaps reflecting the increased 3′-UTR binding of the DDX3X mutant (Figure 6H).

To reveal possible biological pathways regulated by DDX3X rG4-binding, we selected mRNAs with peaks in the top quartile containing a G4 (G2-L12)_4_ motif with a logFC > 1 when compared to the mutant DDX3X iCLAE signal (*P* < 0.05). This generated a list of 104 DDX3X target mRNAs (Supplementary Table S6). When the binding of WT and RG-mutant DDX3X to 5′-UTRs of mRNAs of several cancer-related genes was compared, a clear reduction in signal could be seen in the mutant condition (Figure 6J and Supplementary Figure S7B). Gene ontology enrichment analysis assigned 84 transcripts to specific terms/pathways including adenosine triphosphate maintenance and mitochondrial membrane integrity terms (Supplementary Figure S7C). It was evident that DDX3X binds to several mRNAs that encode components of the oxidative phosphorylation system, suggesting a role of DDX3X in the regulation of energy production in the cell (Supplementary Figure S7D). Overall, these findings suggest that WT DDX3X binds a subset of mRNA targets through rG4 recognition mediated by its GAR domain and that this interaction is notable for components of the oxidative phosphorylation machinery.

## DISCUSSION

A greater understanding of the roles of rG4 structures in mRNA post-transcriptional control will be achieved through a comprehensive knowledge of associated proteins. Over 1500 human RBPs have been cataloged (31,48), with most having no assigned role. Affinity selection has defined sequence motifs for only ∼200 RBPs (49), so it remains a critical question whether RNA secondary structure or the sequence *per se*, is pivotal for RNA–RBP interaction. As a step towards this, we have developed an unbiased AE-LC-MSMS approach to identify cytoplasmic RBPs that interact with the NRAS 5′-UTR rG4 structure. The largest category of rG4 interactors consisted of proteins involved in RNA splicing and processing, followed by proteins involved in translation. Curiously, rRNA base methylation was revealed as a significant term (*P*-value < 0.0005), which may be important since FMRP binding sites are enriched in 6mA-methylation at rG4 motifs (50). Another example of a regulatory RBP–rG4 epigenetic interaction is seen with the polycomb repressive complex (PRC2), which recognizes rG4s in histone-associated RNAs to promote epigenetic silencing (51).

It is noteworthy that we identified several helicases as significant rG4 interactors. This supports the view that there is a dynamic balance between forming and resolving rG4 structures. It has been suggested that in cells rG4s are globally unfolded, which is mediated by rG4 interacting proteins (6). However, these conclusions are drawn from transcriptome-wide averaging and may obscure the dynamics of rG4 formation in individual transcripts. Within the helicases, eIFA4I was not observed as a specific rG4 binder (Figure 3B and Supplementary Table S1). This contrasts with earlier work combining ribosome foot-printing with eIF4AI inhibition which suggested a link between rG4s and eIF4AI (19). A possible explanation is that rG4 association with eIF4AI can only be detected under conditions when eIF4AI is hampered by small molecules and is part of the translation initiation complex eIF4F (19,52).

GAR domains are commonly found in RBPs including several known rG4-interacting proteins (39,53–55). Here, we have extended the number of rG4 interactors that contain a GAR domain and we have confirmed that rG4-binding was abrogated by mutation of this domain for DDX3X and DDX17. Our work and the work of others (56) has revealed several rG4-interacting proteins, including GRSF1 and NSUN5, that do not possess a GAR domain. This might point to the existence of two classes of rG4-interacting proteins, one dependent on the presence of the GAR domain and another class possessing alternative RNA-binding modes specialized for rG4 recognition.

The main focus of our study was identification of rG4-dependent mRNA binders in the cytoplasm. Hence, cytoplasmic extracts rather than total cell extracts were used. Indeed, we could determine several new rG4-binding proteins but also proteins that had been detected in rG4-dependent AEs from total cell extracts, such as GRSF1 and NSUN5 (56). While GRSF1 is a protein targeted to mitochondria, NSUN5 is considered a nuclear protein. Our targeted approach, with focus on cytoplasmic events, now suggests NSUN5 as a potential shuttling protein that might have roles in methylation of mRNAs in the cytoplasm. For future experiments, it is important to study localization of rG4-binding proteins in regards to their molecular function.

Unraveling the function of rG4 interactors also requires identification of their mRNA targets. We were particularly interested in rG4 structures as recognition elements for rG4s-binding proteins. To address this, we compared WT DDX3X to RG-mutated DDX3X iCLAE data. The analysis of DDX3X iCLAE experiment uses stringent log-fold change cut-off of one to generate Supplementary Table S6 which shows for the first time that DDX3X, an important cancer-related helicase, has a set of RNA targets that require an rG4 structure for recognition. However, using these stringent restrictions might prevent detection of other mRNA targets. For instance, the iCLAE experiment detects peaks over the NRAS 5′-UTR as shown in Figure 6J and listed in Supplementary Table S5 and a decrease in DDX3X binding upon mutation of the GAR domain is evident. Still, this decrease does not meet the stringent cut off log-fold change cut-off of one (Log2_fold for NRAS - 0.65).

It is noteworthy that we uncovered a cluster of DDX3X targets that encode proteins involved in the oxidative phosphorylation chain. Indeed, dysregulation of the synthesis of oxidative phosphorylation components has severe consequences and is linked to several diseases, including Huntington's, Alzheimer’s, Parkinson’s disease (57) and cancer (58).

In summary, we have identified new cytoplasmic RBPs that interact with the rG4 secondary structure. The majority of rG4 binders contain GAR domains and mutation of this domain in the clinically important rG4-interacting protein DDX3X hampered the interaction *in vitro* and in cells. Moreover, we discovered that DDX3X mRNA targets are significantly enriched in rG4s with most of the top 104 mRNAs encoding for essential components of the mitochondrial oxidative phosphorylation chain. The discovery of rG4-interacting proteins will enable future mechanistic studies of rG4 dynamics and function in the cell.

## DATA AVAILABILITY

RNA-sequencing and iCLAE data have been deposited at Gene Expression Omnibus (GEO) (GSE106476). The AE-LC-MSMS data have been deposited to the ProteomeXchange Consortium via the PRIDE partner repository with the dataset identifier PXD010860.

## Supplementary Material

Supplementary Data
