# Supplementary material for: Analysis of NRAS RNA G-quadruplex binding proteins reveals DDX3X as a novel interactor of cellular G-quadruplex containing transcripts
Source: Nucleic Acids Res. 2018 Sep 26;46(21):11592–604. doi: 10.1093/nar/gky861 (PMC6265444; doi:10.1093/nar/gky861)
Supplement: Supplementary Data [file gky861_supplemental_files.zip › Supplementary Figures.pdf]

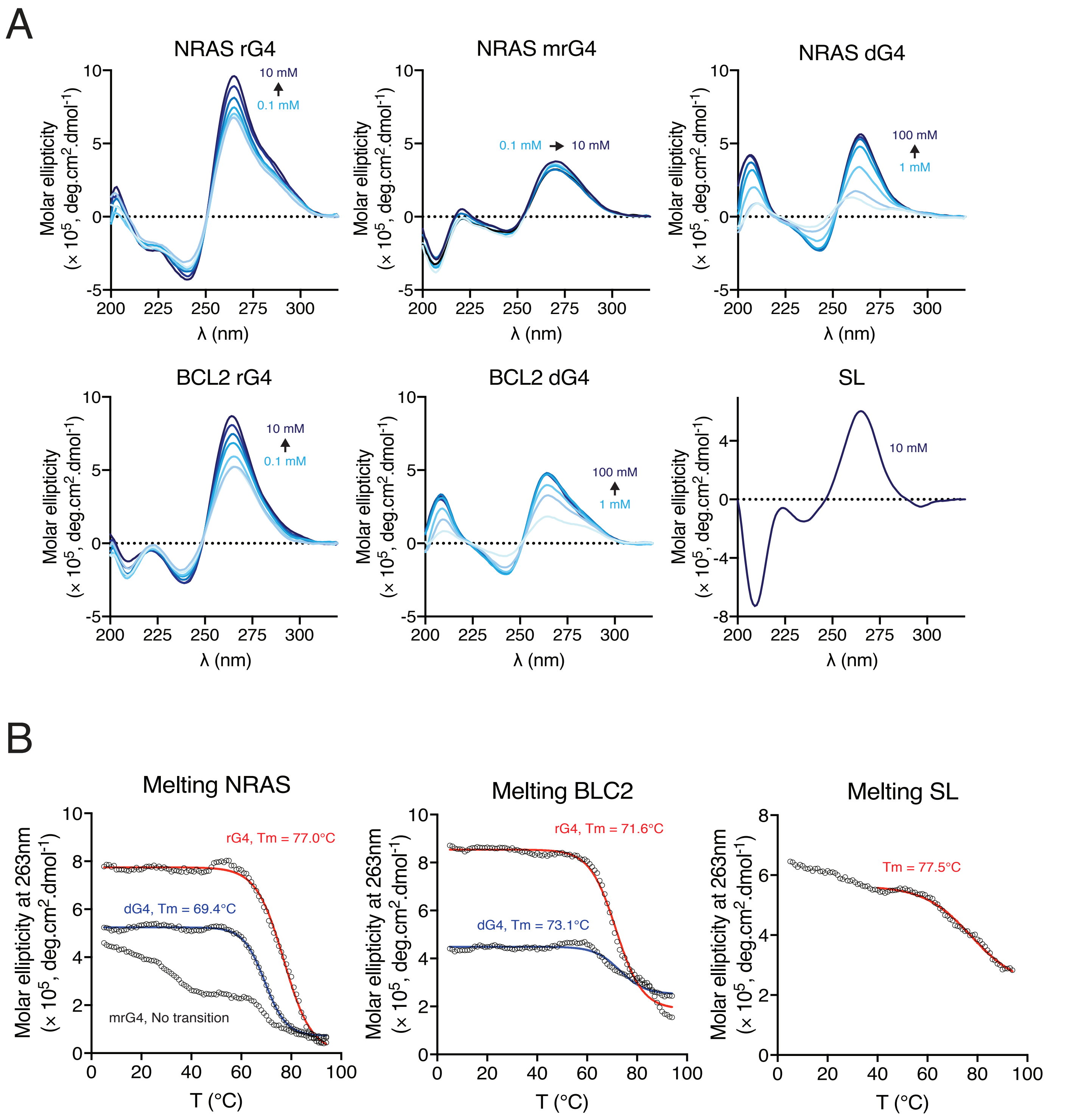

Supplementary Figure 1

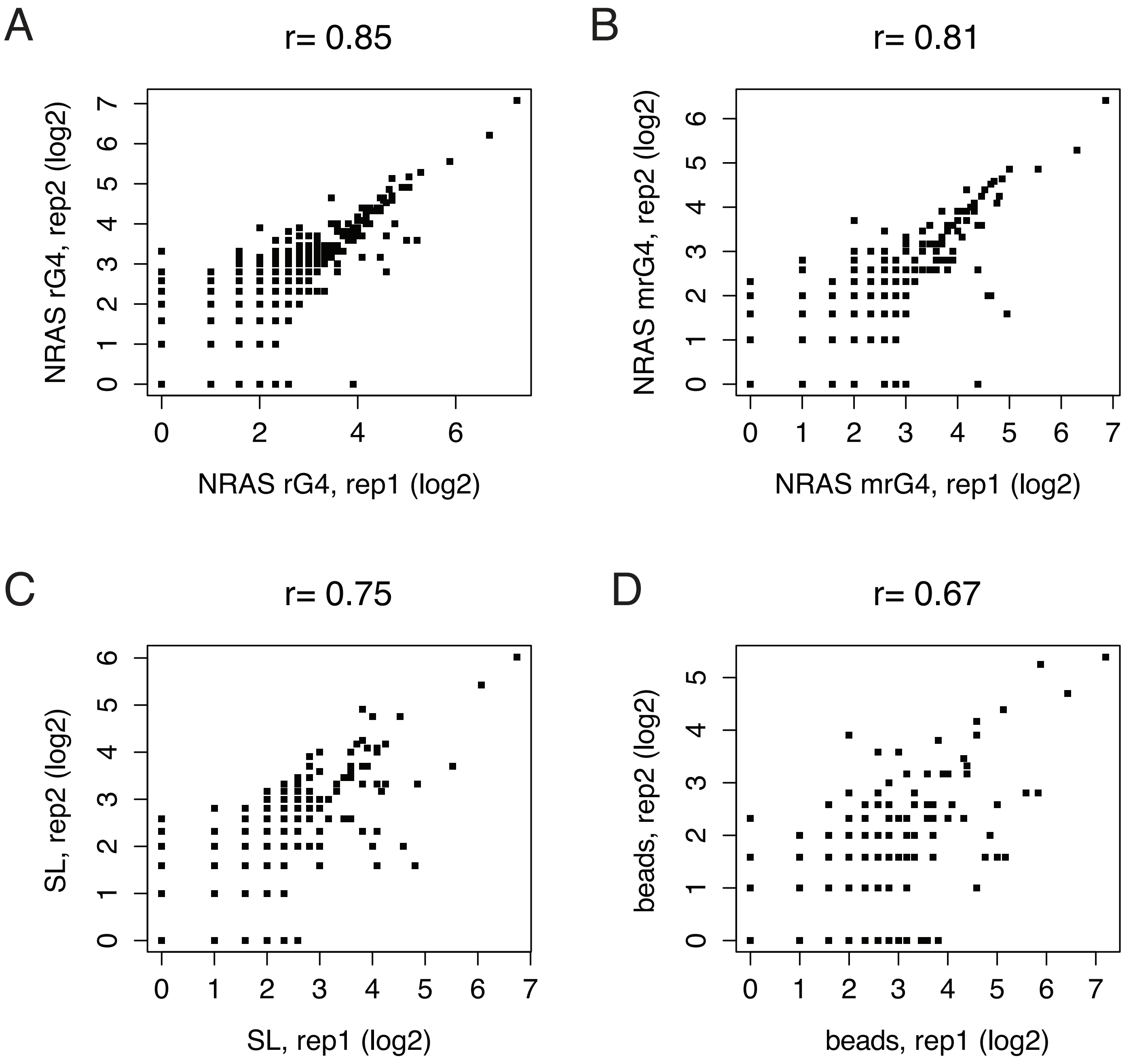

Supplementary Figure 2

A

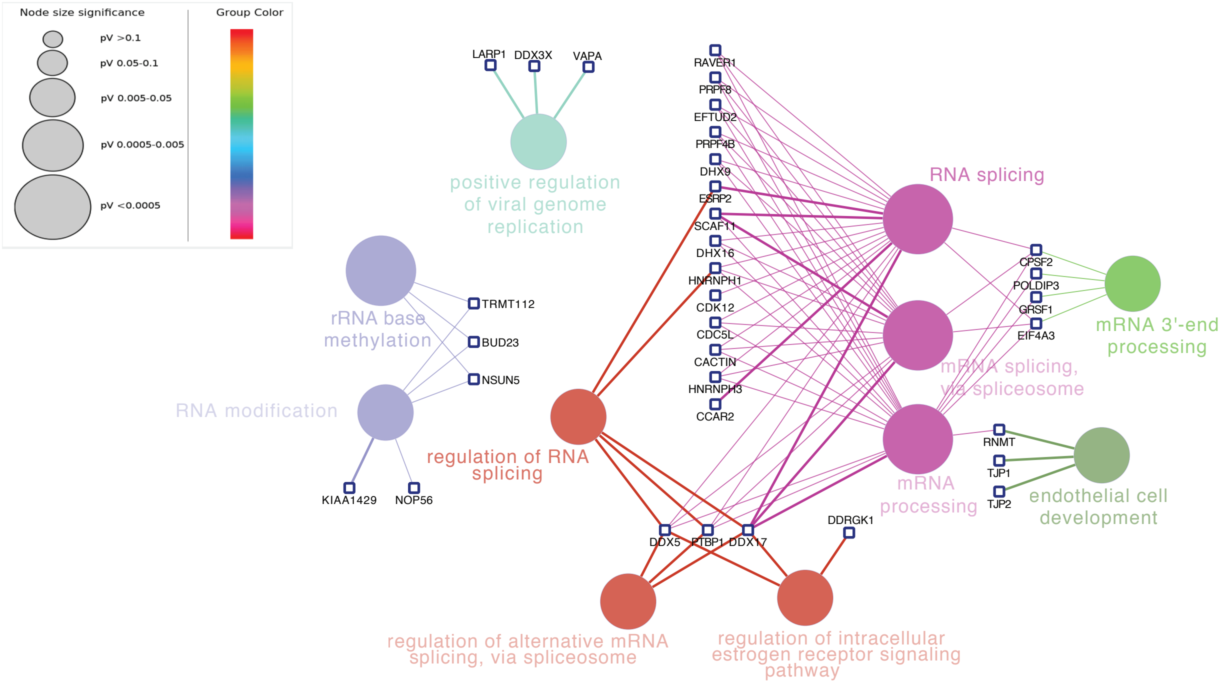

Supplementary Figure 3

A

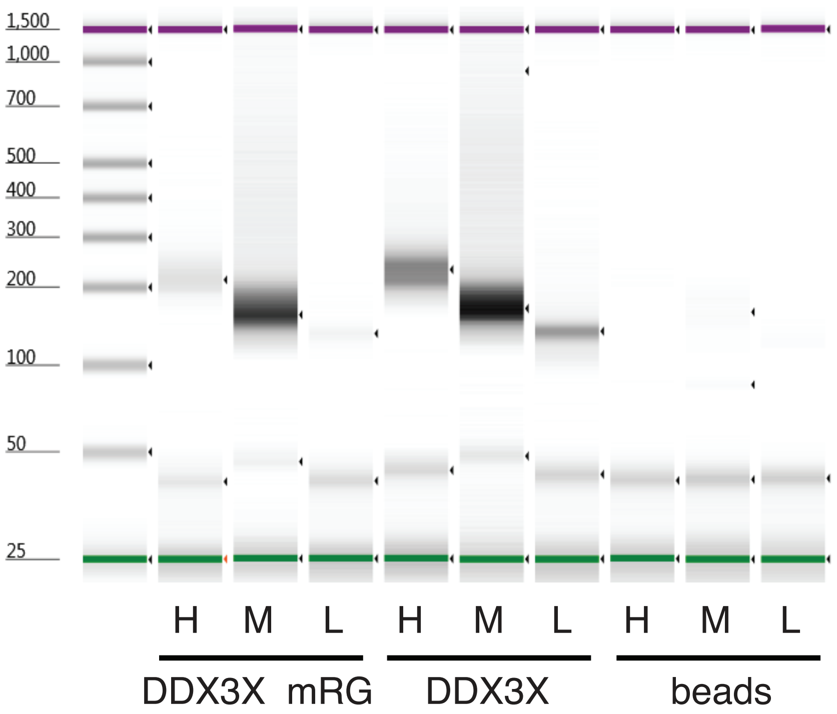

Supplementary Figure 4

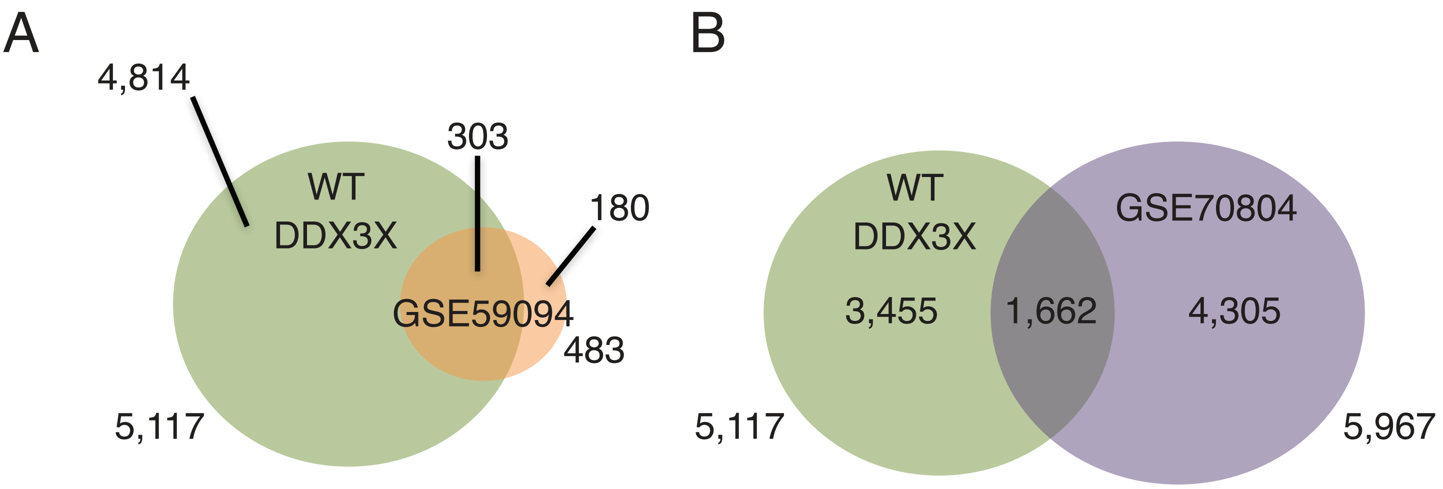

C

Overlap with published data sets								
Peak set	Peaks in transcripts	Intersection	Random shuffling #1	Random shuffling #2	Random shuffling #3	Average random	Average random	SD fold
WT	5,117	303	9	11	9	9.7	31.3	0.119
GSE59094	483							
WT	5,117	1662	33	44	31	36	46.2	0.194
GSE70804	5,967 (23% G2L12)							

Supplementary Figure 5

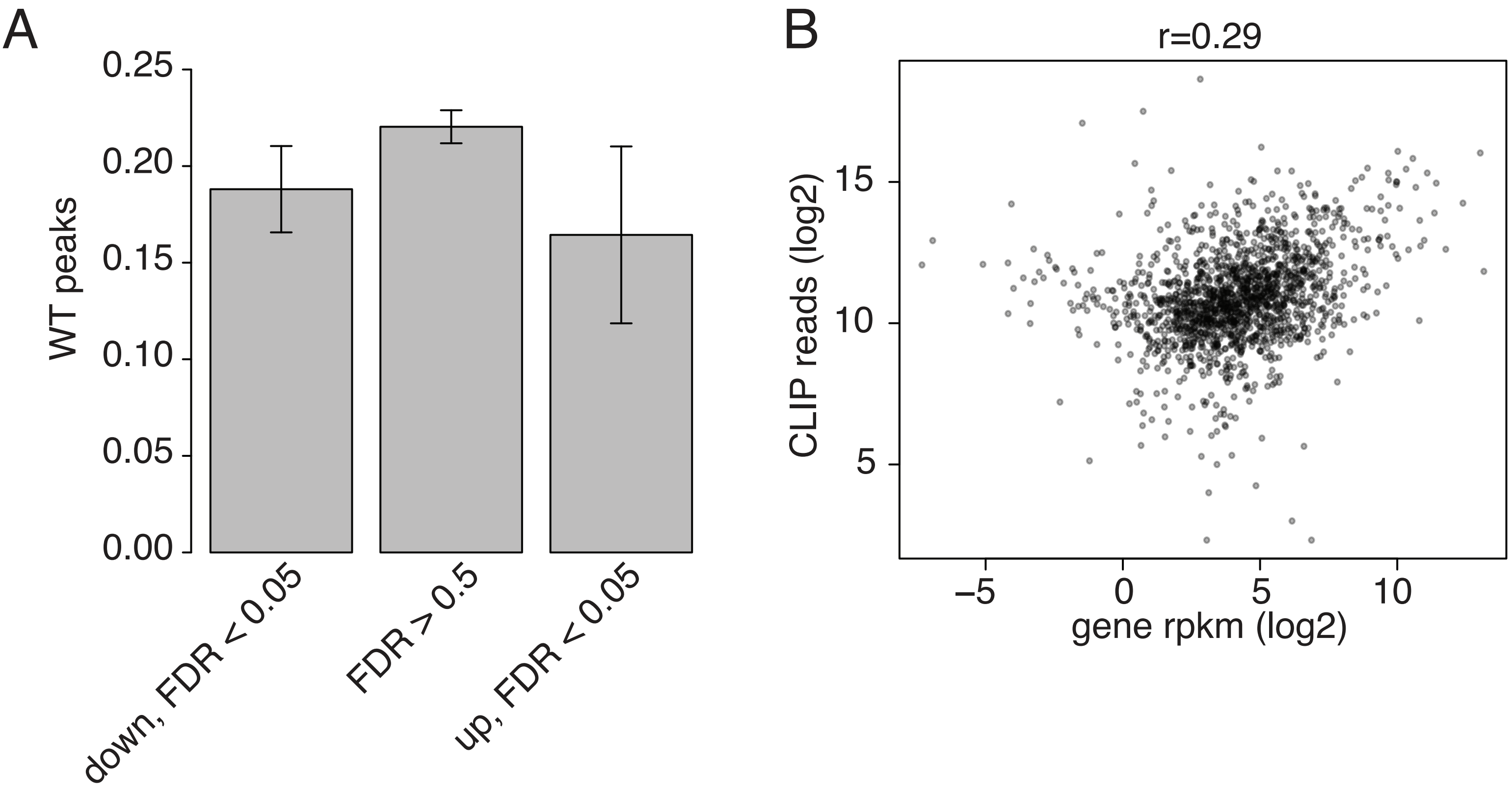

Supplementary Figure 6

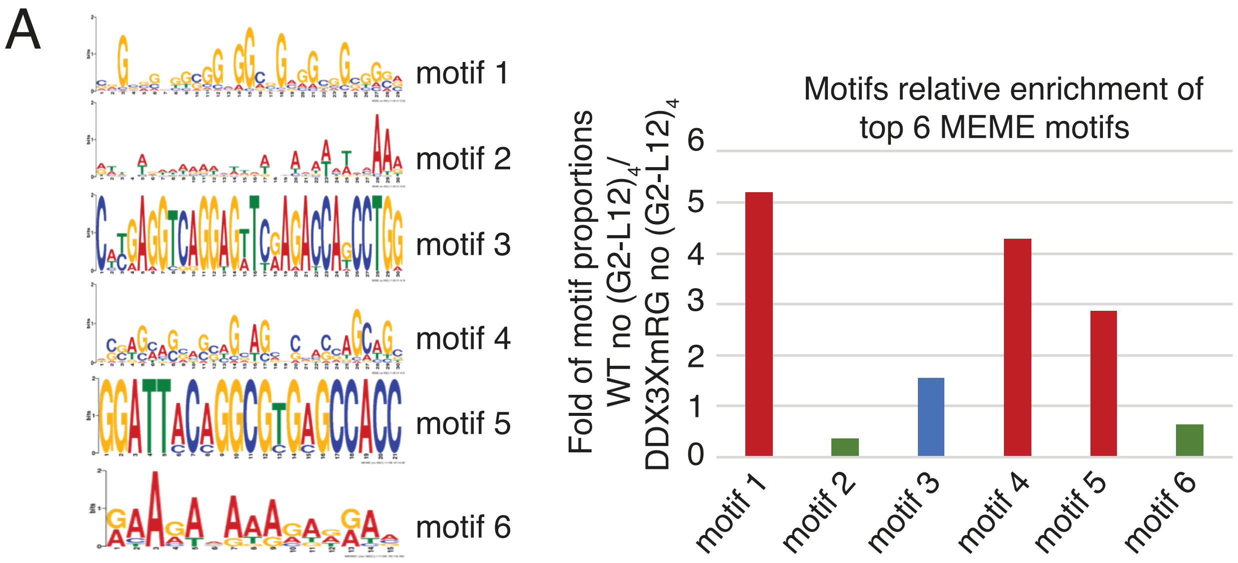

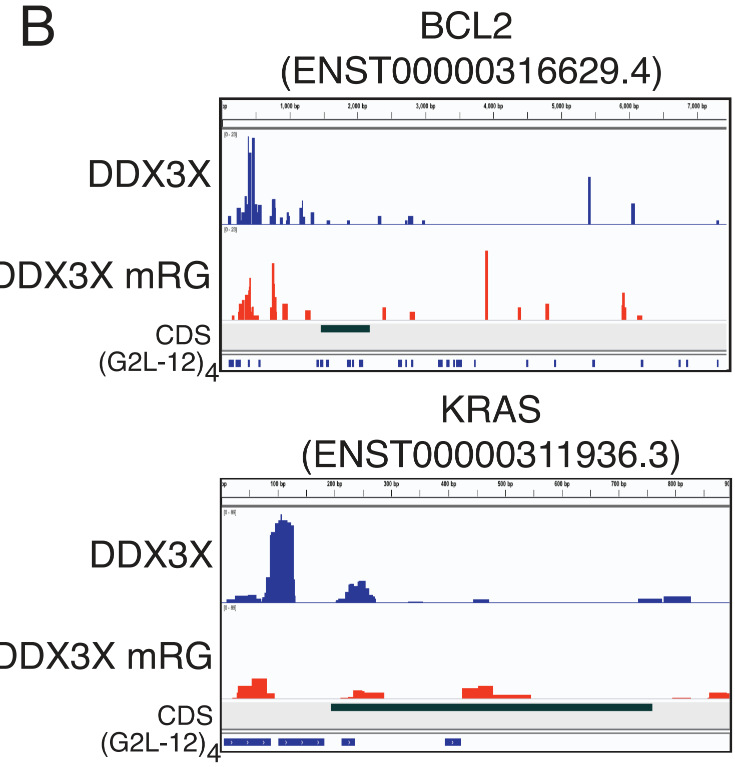

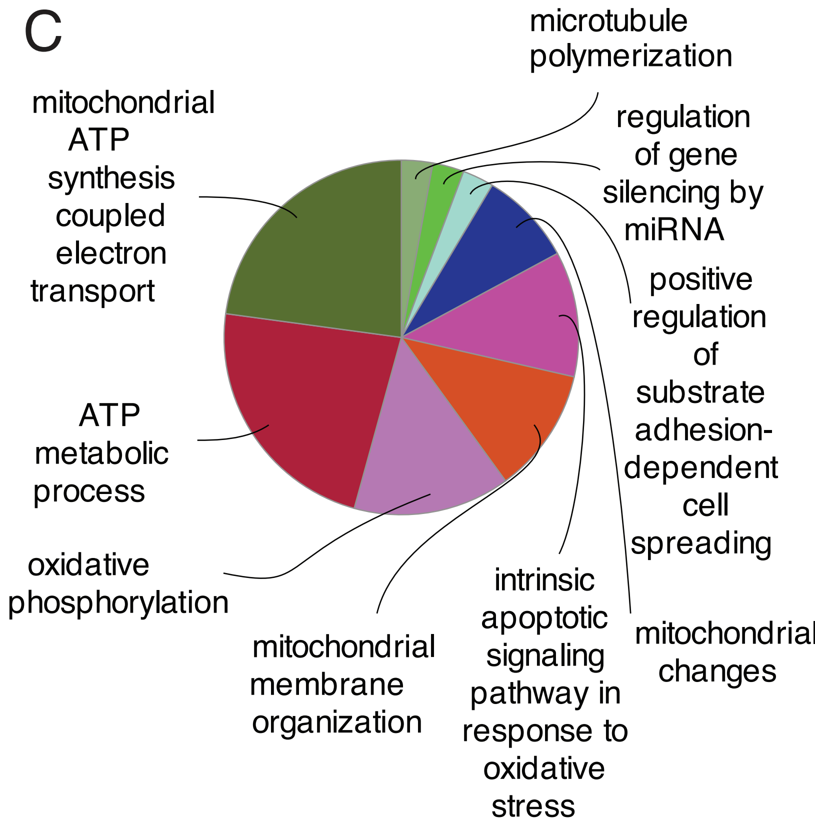

D

Protein	OXPHOS complex	Protein	OXPHOS complex
NDUFAB1	I	QCR8	III
NDUFB7	I	COX6A	IV
NDUFB10	I	COX6B	IV
ISP	III		

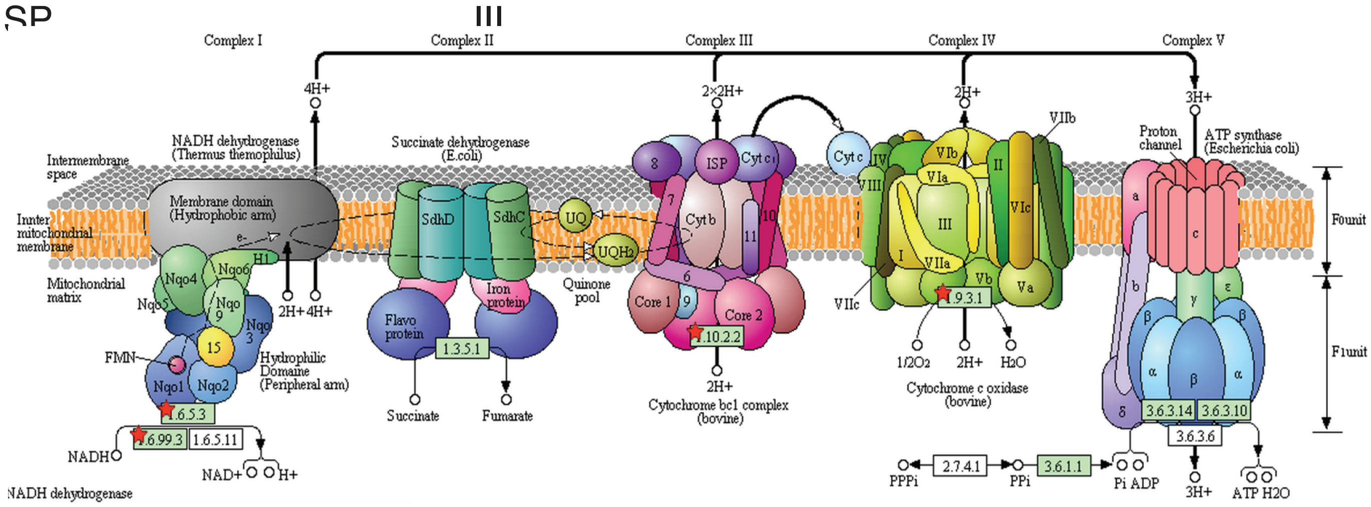

Supplementary Figure 7
