# Supplementary material for: Analysis of NRAS RNA G-quadruplex binding proteins reveals DDX3X as a novel interactor of cellular G-quadruplex containing transcripts
Source: Nucleic Acids Res. 2018 Sep 26;46(21):11592–604. doi: 10.1093/nar/gky861 (PMC6265444; doi:10.1093/nar/gky861)
Supplement: Supplementary Data [file gky861_supplemental_files.zip › Supplementary Information.docx]

**SUPPLEMENTAL INFORMATION TITLES AND LEGENDS**

**SUPPLEMENTAL EXPERIMENTAL PROCEDURES**

**Circular Dichroism and UV thermal melting analysis.** Experiments were conducted as previously described (Guilbaud et al., 2017). Briefly, oligonucleotide solutions were prepared at a final concentration of 10 µM in 10 mM lithium cacodylate (pH 7.2) containing 1mM EDTA and titrations were performed by adding potassium chloride. Scans were measured over the range of 200–320 nm at 20 °C. Melting studies were performed using KCl concentrations of 10 and 100 mM for RNA and DNA samples respectively. The CD signal at 263 nm was monitored and melting temperature (Tm) values were extracted as the half-maximum decrease in ellipticities.

**SUPPLEMENTAL FIGURES**

**Supplementary Figure 1.** Biophysical analysis of biotinylated RNA oligonucleotides. **A.** Circular dichroism (CD) spectra of indicated oligonucleotides. Parallel G-quadruplexes display a characteristic positive and negative peak at 260 nm and 240 nm respectively. **B.** UV thermal melting curves of indicated oligonucleotides.

**Supplementary Figure 2.** Reproducibility of replicates. **A-D.** Scatter blots of the correlation between peptide counts among replicates for NRAS rG4 AEs and controls (r, Pearson’s coefficient). Each dot represents one protein with at least one peptide count (n) in both replicates. **A.** NRAS rG4 ( n=815), **B.** NRAS mutated rG4 (mrG4) (n=521), **C.** stem loop (SL) (n=437) and **D.** beads (n=357) replicates are compared.

**Supplementary Figure 3.** Key roles of high confidence NRAS rG4 interactors in essential pathways regulating mRNA fate. **A.** Gene set enrichment analysis using the Cytoscape app CluGo. Enriched GO pathways are shown as functionally grouped network. Terms represent nodes linked by edges based on their kappa score (≥0.3). Only the label of the most significant term per group is shown. The node size represents the significance of the enriched GO term. Functionally related groups partially overlap.

**Supplementary Figure 4.** Libraries prepared from DDX3X iCLAE. H, M and L indicate the

high medium and low fractions isolated from cDNA as per the iCLIP protocol (Huppertz et al, 2014). **A.** 50% more cells were used to obtain libraries for the GAR mutant DDX3X (DDX3X mRG). No library could be amplified from the bead only fractions (Vec).

**Supplementary Figure 5.** Overlap with published data sets. Venn diagrams showing overlaps of WT DDX3X binding regions and those previously described in **A.** GSE59094 (Valentin-Vega et al., 2016) and **B.** GSE7080 (Oh et al. 2016). **C.** Table summarizing the intersection and the overlap obtained after random reshuffling of binding regions in transcript regions only. The likelihood over a random overlap is depicted in fold enrichment. High confidence binding regions were identified by overlapping peaks in at least 2 replicates in each data set.

**Supplementary Figure 6.** Relation between RNA-seq and iCLAE data sets. **A.** Barplot showing the average peak number of identified DDX3X wild type (WT) peaks in relation to differentially expressed genes. Differences are not significant (p > 0.1) . **B.** Scatter plot of the correlation between read count in the RNA-seq experiment, expressed as log2 of the RPKM, and iCLAE reads in the same gene (r = Pearson correlation coefficient). Both plots display low dependency of mRNA abundance on iCLAE binding regions.

**Supplementary Figure 7.** mRNAs identified by DDX3X iCLAE constitute a post-transcriptional operon potentially modulating the oxidative phosphorylation system (OXPHOS). **A.** Gene browser view of selected DDX3X interacting mRNAs **B.** CluGo enrichment of terms associated with distinct regulatory pathways. **C.** MEME motive-based sequence analysis of mRNAs bound by DDX3X that do not contain an rG4 as defined by (G2-L12)_4_. Analyzed were 1,048 WT_no_(G2-L12)_4_ peaks out of 2,449 total peaks and , 1383 peaks DDX3X mRG_no_(G2-L12) out of 1,785 total mutant peaks. Six motifs were enriched and FIMO was used to calculated the number of peaks within each given motif. **D.** List of identified DDX3X, rG4 containing mRNAs that encode proteins present in the several complexes of the OXPHOS system. For orientation a cartoon of the OXPHOS system was added from the KEEG pathway database. Red stars indicate the position of several proteins encoded by rG4 containing DDX3X target mRNAs .

**SUPPLEMENTARY TABLES**

**Supplementary Table 1.** Results of AF-MS AEs of proteins interacting with the human NRAS rG4 structure and information about RG/RGG domains in identified proteins. Columns G4_A, G4_B, mG4_A, mG4_B, SL_A, SL_B, B_A and B_B show peptide counts of indicated conditions in the “All_Information” sheet. The “FDR.G4_vs_CTRs” column, represents the computed false discovery rate (FDR) comparing the peptide counts NRAS rG4 AEs with controls (mNRAS rG4, SL and beads). FDR of 0.05 was used as a cut off.

**Supplementary Table 2.** Refinement of AF-MS results. Identified proteins were ranked according to their FDR (significant FDR < 0.05). This revealed 80 significant rG4 interactors. This list was further shortened by only considering proteins detected by 6 or more unique peptide counts (UPC) (FDR < 0.05 UPC ≥ 6).

**Supplementary Table 3.** Information about cloned rG4 interacting proteins. cDNA source is described and the positions of mutated GAR domains are indicated.

**Supplementary Table 4.** List of primers used to establish gateway compatible entry clones and GAR domain mutations. Name of rG4 interactor and sequences of forward (fw) and reverse (rv) primer are displayed.

**Supplementary Table 5.** Transcripts interacting with DDX3X determined by iCLAE. Consensus peaks were identified in two out of three biological replicates for WT DDX3X and aligned to the transcriptome. P-values were calculated by fitting the data to a linear model.

**Supplementary Table 6.** List of top rG4 containing transcripts interacting with specifically with DDX3X. Transcripts with peaks in the top quartile containing the G4 (G2-L12)4 motif with a logFC > 1 when compared to the mutant DDX3X iCLAE signal (p < 0.05) were selected.
